# From Neural Crest Development to Cancer and Vice Versa: How p75^NTR^ and (Pro)neurotrophins Could Act on Cell Migration and Invasion?

**DOI:** 10.3389/fnmol.2018.00244

**Published:** 2018-08-23

**Authors:** Sabine Wislet, Geoffrey Vandervelden, Bernard Rogister

**Affiliations:** ^1^GIGA-Neurosciences, University of Liège, Liège, Belgium; ^2^Department of Neurology, University of Liège, Liège, Belgium

**Keywords:** p75^NTR^, neurotrophin, migration, invasion, cadherin, ephrin, kidins220

## Abstract

The p75 neurotrophin receptor (p75^NTR^), also known as low-affinity nerve growth factor, belongs to the tumor necrosis factor family of receptors. p75^NTR^ is widely expressed in the nervous system during the development, as well as, in the neural crest population, since p75^NTR^ has been described as ubiquitously expressed and considered as a neural crest marker. Neural crest cells (NCCs) constitute an transient population accurately migrating and invading, with precision, defined sites of the embryo. During migration, NCCs are guided along distinct migratory pathways by specialized molecules present in the extracellular matrix or on the surfaces of those cells. Two main processes direct NCC migration during the development: (1) an epithelial-to-mesenchymal transition and (2) a process known as contact inhibition of locomotion. In adults, p75^NTR^ remains expressed by NCCs and has been identified in an increasing number of cancer cells. Nonetheless, the regulation of the expression of p75^NTR^ and the underlying mechanisms in stem cell biology or cancer cells have not yet been sufficiently addressed. The main objective of this review is therefore to analyze elements of our actual knowledge regarding p75^NTR^ roles during the development (mainly focusing on neural crest development) and see how we can transpose that information from development to cancer (and vice versa) to better understand the link between p75^NTR^ and cell migration and invasion. In this review, we successively analyzed the molecular mechanisms of p75^NTR^ when it interacts with several coreceptors and/or effectors. We then analyzed which signaling pathways are the most activated or linked to NCC migration during the development. Regarding cancer, we analyzed the described molecular pathways underlying cancer cell migration when p75^NTR^ was correlated to cancer cell migration and invasion. From those diverse sources of information, we finally summarized potential molecular mechanisms underlying p75^NTR^ activation in cell migration and invasion that could lead to new research areas to develop new therapeutic protocols.

## Introduction

The p75 neurotrophin receptor (p75^NTR^) was first identified in Sutter et al. (1979) and described as a low-affinity nerve growth factor (NGF) receptor. In the early eighties, p75^NTR^ was already described as significantly overexpressed in multiple neural crest-derived cancers such as melanoma or neurofibroma (Ross et al., 1984); however, its role in normal conditions was still undefined even if its distribution during the development started to be well characterized. Indeed, in 1990, Heuer and collaborators described the expression of low-affinity NGF receptors in premigratory neural crest cells (NCCs), in epibranchial placode cells, and in all sensory, sympathetic, and parasympathetic derivatives of these structures. In the central nervous system, at the later stage of the development, p75^NTR^ was detected in a substantial fraction of cells in every brain region, with the highest levels present in developing motor neurons (Heuer et al., 1990). Finally, p75^NTR^ was also observed in mesenchymal cell populations including cells in branchial arch, sclerotome, muscle, and feather follicles (Heuer et al., 1990). Baldwin et al. (1992) described the structure of p75^NTR^ containing an extracellular region with four cysteine-rich repeat regions (loops), all of which were required for ligand binding. Since then, more than 10,000 studies have been conducted to understand and characterize the roles of this receptor, in normal and pathological conditions. In 2018, new roles for this receptor are still suggested in cancer cell invasion and migration. Indeed, migration is defined as a process by which a cell goes from one point to another adopting several motility modes, whereas invasion characterizes a cell’s ability to become mobile and migrate through a tissue or infiltrate neighboring tissues. Currently, p75^NTR^ expression has been correlated to cell migration and invasion in multiple cancer types; however, the molecular pathways underlying p75^NTR^ roles, in those processes, remain unclear. Therefore, the main objective of this review is to analyze all information regarding p75^NTR^ roles in migration and invasion, during the development (mainly focusing on NCCs) and potentially transpose the information to the observations made in cancers (and vice versa). To do so, we will first summarize our actual knowledge regarding p75^NTR^ structure and binding partners with regard to migration and invasion processes. We will then deeply analyze the molecular pathways underlying p75^NTR^ roles in NCC invasion and migration during the development and compare those migration and invasion molecular pathways to the information related to cancer cell migration and invasion. Altogether, this will provide us a clearer view of the main factors regulating p75^NTR^ functions in cell migration and invasion, thereby facilitating the development of new therapeutic strategies in cancer treatments.

## Structure of p75^NTR^ and Binding Partners

**FIGURE 1:**
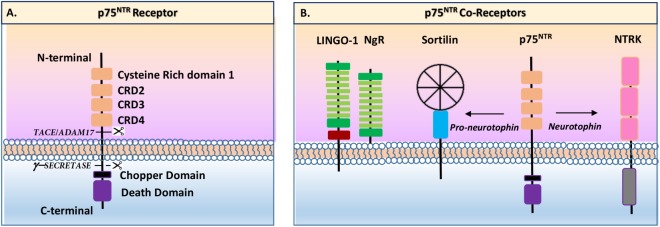
Schematic view of p75^NTR^ receptor and coreceptors (inspired by Skeldal et al., 2011). **(A)** p75^NTR^ present an extracellular N-terminal part including four cysteine-rich domains (CRD) and a single-peptide transmembrane domain, and an intracellular c-terminal part containing a Chopper domain and a death domain. The extracellular part is cleavable by TACE/ADAM17 enzyme while the intracellular part is cleavable by gamma-secretase enzyme. **(B)** p75^NTR^ has several coreceptors including sortilin, TrkA, TrkB, TrkC, and Nogo receptors (NgR).

p75^NTR^ is a single membrane-spanning protein in the tumor necrosis factor (TNF) receptor family (Heuer et al., 1990). p75^NTR^ is a 427-amino-acid transmembrane receptor containing an extracellular domain (ECD) with a cleavable 28 amino acid (aa) signal peptide, followed by four 40 aa extracellular cysteine-rich domains, one of which contains a *N*-glycosylated site (**Figure 1A**). Multiple *O*-glycosylation sites exist in the juxtamembrane domain. The intracellular domain (ICD) contains an 80 aa death domain (DD), the signature of the tumor necrosis factor (TNF) receptor family that includes sites for interactions with numerous signaling effectors. The DD is a globular complex protein including six-helix bundle fold. DD-containing proteins play important roles in inflammatory and in apoptotic signaling through the formation of oligomeric protein complexes (Lin et al., 2015). p75^NTR^ contains a DD similar, in some ways, to other TNF-associated DDs. However, a major difference between p75^NTR^ and other TNF receptors is that the p75^NTR^ ICD is unable to autoactivate and has to interact with other effector proteins to exert its functions (Meeker and Williams, 2014). Another remarkable specificity of p75^NTR^ (compared to other TNF receptors) is the 29 AA juxtamembrane region, which is also known as the Chopper domain, located at the N-terminal part of the DD. In this case, the Chopper domain has the ability to independently interact with apoptotic peptidase-activating factor 1 (APAF-1) to induce cell death (reviewed by Skeldal et al., 2011; Meeker and Williams, 2014). Altogether and depending on the type of interactions with a specific coreceptor and/or effector, a wide range of signaling events may occur that lead to many different responses (Meeker and Williams, 2014). The most significant identified p75^NTR^ signaling pathways include Ras homolog gene family, member A (RhoA), Jun N-terminal kinase (JNK), mitogen-activated protein kinase (MAPK), and nuclear factor κappa B (NFκB) (Wang et al., 2008). These pathways are possibly activated through various regions of the ICD upon interactions with other proteins (Wang et al., 2008). Among those proteins, guanine nucleotide dissociation inhibitor (Rho-GDI), ribosome-inactivating protein-2 (RIP-2), and p75^NTR^-associated cell death executor (NADE) have been described as interacting with the p75^NTR^ DD (Wang et al., 2008). Likewise, Schwann cell factor-1 (SC-1), neurotrophin receptor-interacting MAGE homolog (NRAGE), tumor necrosis factor (TNF) receptor-associated factor (TRAF), and neurotrophin receptor-interacting factor (NRIF) have been described as inducing those signaling pathways through interactions with the juxtamembrane region of p75^NTR^ (Wang et al., 2008). Finally, it was demonstrated that an ankyrin-rich membrane-spanning protein (ARMS) initially identified as Kidins220 is able to interact with the ICD to form a ternary complex with Trk receptors (Chang et al., 2004).

Beside intracellular effectors, neurotrophins (NTs) play pivotal roles in p75^NTR^ signaling pathways. NTs belong to a large group of growth factors that mainly target neurons of the central and peripheral nervous systems, during the development. NTs are known, among other activities, to stimulate neurite growth, to maintain viability, or to induce cell differentiation. The first NT to be defined was the NGF (Sutter et al., 1979). Historically, it was also the first growth factor to be purified and characterized in 1960 (Aloe, 2004). Several years later, a homolog of the NGF was isolated, characterized, and named brain-derived neurotrophic factor (BDNF) (Barde et al., 1982). Molecular cloning experiments defined two more mammalian members of this family, NTs 3 and 4/5 (NT3 and NT4/5) (Maisonpierre et al., 1991; Ip et al., 1992). NTs activate two different classes of receptor: the tropomyosin-related kinase receptors family (Trk A, B, and C) and the low-affinity-NGF receptor named p75^NTR^ (Huang and Reichardt, 2001).

Neurotrophins are expressed by the cells as a proneurotrophin (proNT) and are matured by proteolysis processes. When proNTs were initially discovered, they were considered as inactive precursors of the mature proteins (reviewed by Meeker and Williams, 2014). However, multiple studies indicated that the proNTs are also physiologically active (Lee et al., 2001) and that NT signaling pathways work through a balance between mature and immature (pro-) NTs (reviewed by Meeker and Williams, 2014). Likewise, it has been suggested that the interactions of p75^NTR^ with its coreceptors regulate the availability of high-affinity targets for mature or immature (pro-) NTs (reviewed by Meeker and Williams, 2014).

Neurotrophins can act directly or indirectly on p75^NTR^ mainly through its interactions with three major coreceptor families: Trk (A, B, and C), sortilin, and Nogo receptors (Meeker and Williams, 2014; **Figure 1B**). Trk receptors were first identified as colon-derived oncogene in which tropomyosin was fused to a tyrosine kinase domain. Currently, three Trk receptors have been identified: TrkA (or NTRK1), TrkB (or NTRK2), and TrkC (or NTRK3). Trk receptors are characterized by the presence of an extracellular domain, with three tandem leucine-rich motifs flanked by two cysteine clusters in their amino termini, and two immunoglobulin-like domains in the membrane-bound region (Huang and Reichardt, 2001). Differential splicings of the TrkA, TrkB, and TrkC mRNAs result in protein expression with various ligand affinities due to changes into their extracellular domain (Huang and Reichardt, 2001). The presence or absence of short amino-acid sequences, in the juxtamembrane domain of each receptor, affects the ability of some NTs to activate these receptors (Huang and Reichardt, 2001). Each Trk has therefore a different NT binding specificity: TrkA binds NGF, TrkB binds BDNF and NT4/5, and TrkC binds NT3 (TrkA and TrkB bind NT3 but to a lesser extent). As reviewed by Pramanik et al. (2016), interactions between Trk receptors and p75^NTR^ increase the binding affinity for NTs and support pro-survival and pro-growth signaling via various pathways such as MEK/ERK, PI3K/AKT, and PLCγ. Regarding migration and invasion (and among other examples), TrkA overexpression has been linked to breast cancer cell migration and invasion through PI3K and ERK/p38 MAP kinase pathways (Lagadec et al., 2009). TrkB overexpression has been shown to induce epithelial-to-mesenchymal transition (EMT), leading to invasion processes of head and neck squamous cell carcinoma (Kupferman et al., 2010). Likewise, BDNF/TrkB have been linked to neuroblastoma cell migration through PI3K/Akt/mTOR and MAPK pathway activations (Hua et al., 2016). TrkC has also been linked to cell migration as TrkC upon NT3 activation leads to adenoid cystic carcinoma cell migration through AKT/ERK pathway activations (Ivanov et al., 2013).

Besides Trk receptors, the most studied non-Trk p75^NTR^ coreceptor is sortilin. Sortilin, also known as neurotensin receptor 3 (NTS3 or NTR3), belongs to the vacuolar protein sorting 10 (Vps10) domain family (Wilson et al., 2014). Sortilin is a sorting protein mainly found within late endosomes derived from the trans-Golgi network. In this context, sortilin has been described as assisting the anterograde trafficking of NT receptors and to control proBDNF secretion (Reviewed by Meeker and Williams, 2014). With regards to invasion and migration, it has been demonstrated that proNGF induced migration in thyroid cancer cells, through p75^NTR^/TrkA/sortilin leading to SRC signaling pathway activation (Faulkner et al., 2018). Likewise, in clear cell renal cell carcinoma, it was shown that proBDNF induced cell migration through p75^NTR^/TrkB/sortilin leading to the activation of AKT and ERK pathways (De la Cruz-Morcillo et al., 2016).

The Nogo receptors (NgR1, NgR2, and NgR3) are glycophosphatidylinositol (GPI)-anchored receptors mainly expressed by neurons, in the central and peripheral nervous systems (He et al., 2003). NgRs are cell-surface receptors presenting a highly conserved eight leucine-rich repeats (LRR) flanked by cysteine-rich LRRNT (leucine-rich repeat N-terminal) and LRRCT (leucine-rich repeat C-terminal) domains, as classically described for the LRR family proteins. As described by Saha and collaborators, the LRR domains are connected to the GPI-anchor for membrane attachment via a “stalk” region (Saha et al., 2014). The LINGO-1 receptor is the functional component of NgR. LINGO-1 contains an LRR domain, an immunoglobulin-like domain, a stalk domain, a transmembrane region, and a short cytoplasmic tail. In the migration context, it seems that Nogo receptors could have inhibitory or inductive effects on cell migration and invasion. Indeed, Jin et al. (2016) demonstrated that Nogo-A was able to inhibit the migration and invasion of human malignant glioma cells via the downregulation of RhoA-cofilin signaling. On the contrary, NgR3 seems to induce nasopharyngeal carcinoma cell migration by activating focal adhesion kinase (FAK) (He et al., 2018).

As the main objective of this review is to understand the role of p75^NTR^ in cell migration and invasion, we will mainly focus the next parts of this review on p75^NTR^ cellular and molecular pathways with regard to those processes.

## Roles of p75^NTR^ in Neural Crest Cell Migration During Development

The NCCs constitute an transient population accurately moving and undergoing a wide dispersion along multiple pathways, invading with precision defined sites of the embryo and differentiating into many derivatives (Zanin et al., 2013). As reviewed by Vega-Lopez et al. (2017), NCCs are generated along the entire length of the anterior–posterior axis, with the exception of the most anterior part of the embryo. NCCs are guided along distinct migratory pathways by specialized adhesion molecules in the extracellular matrix or by molecules on the surface of the cells, in the embryonic periphery (Vega-Lopez et al., 2017). The NCCs that migrate and invade multiple tissues originate from four different segments of the anterior–posterior axis: cranial, vagal, trunk, and sacral (Vega-Lopez et al., 2017).

The cranial NCCs participate in the formation of most of the craniofacial connective tissues and structures including craniofacial nerves, bones, oral muscles, tongue, the dental pulp and periodontal ligament, pigment cells, the choroid, the optic nerve, and the retina (Vega-Lopez et al., 2017). The cardiac NCC participates in heart development, while the vagal neural crest contributes to the enteric ganglia of the gut (Vega-Lopez et al., 2017). Finally, the trunk neural crest also give rise to neurons and glia of the peripheral nervous system, secretory cells of the endocrine system, and pigment cells of the skin (Vega-Lopez et al., 2017). It has also been described that bone marrow-derived NCCs that participate to the hematopoietic niches are also originating from the trunk neural crest (Coste et al., 2015).

In those NCC populations, p75^NTR^ has been described as ubiquitously expressed and considered as neural crest marker (Pan et al., 2016). However, even if p75^NTR^ expression is widely observed from embryonic to adult stage, its role remains unclear. Based on knockout mice invalidated for the p75^NTR^ gene, we learned that Schwann cell migration from the dorsal root ganglia was significantly decreased in the p75^-/-^ embryos (Bentley and Lee, 2000). Based on the same knockout mouse model, Wang and collaborators suggested that p75-dependent signaling plays a crucial role in the migration of epidermal Langerhans cells (LC) and in the initiation of cutaneous immune responses (Wang et al., 1997). Since p75^NTR^ is strongly expressed in migrating NCCs, Kaartinen’s team (Bogenmann et al., 2011) generated tissue-specific p75^NTR^ mutants by crossing p75^NTR-*LOX/LOX*^ mice with transgenic Wnt1-Cre driver mice, which are known to be able to induce a robust recombination in early migratory NCCs (Jiang et al., 2000). According to this study, it appeared that p75^NTR^ was specifically ablated in the dorsal root ganglia, as observed for the full p75^NTR^ KO mice. In the same study, the authors showed a decrease of 30% in the sciatic nerve diameter compared to the control littermates (Bogenmann et al., 2011). Likewise, p75^NTR-*Lox/lox*^/Wnt1-Cre mutants presented a hematopoiesis deficiency (Bogenmann et al., 2011) suggesting a dysregulation of the hematopoietic niches with regards to the potential absence of NCCs in bone marrow, in such transgenic mice (Garcia-Garcia et al., 2015).

**FIGURE 2:**
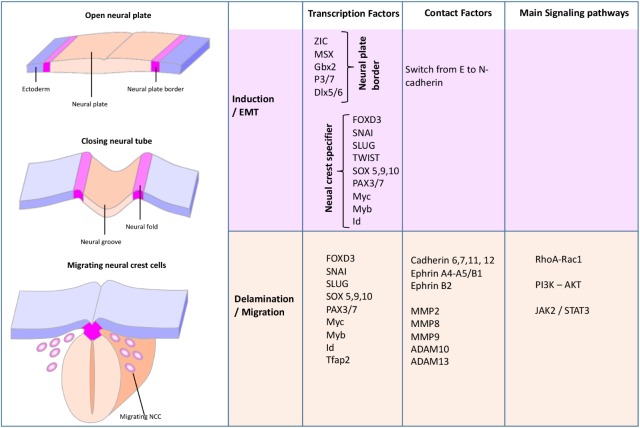
Schematic view of the neural crest formation and migration during the early steps of the development (Glejzer et al., 2011). During the NCC development, several transcription factors, contact factors, as well as signaling pathways orchestrate NCC EMT, invasion, and migration.

**FIGURE 3:**
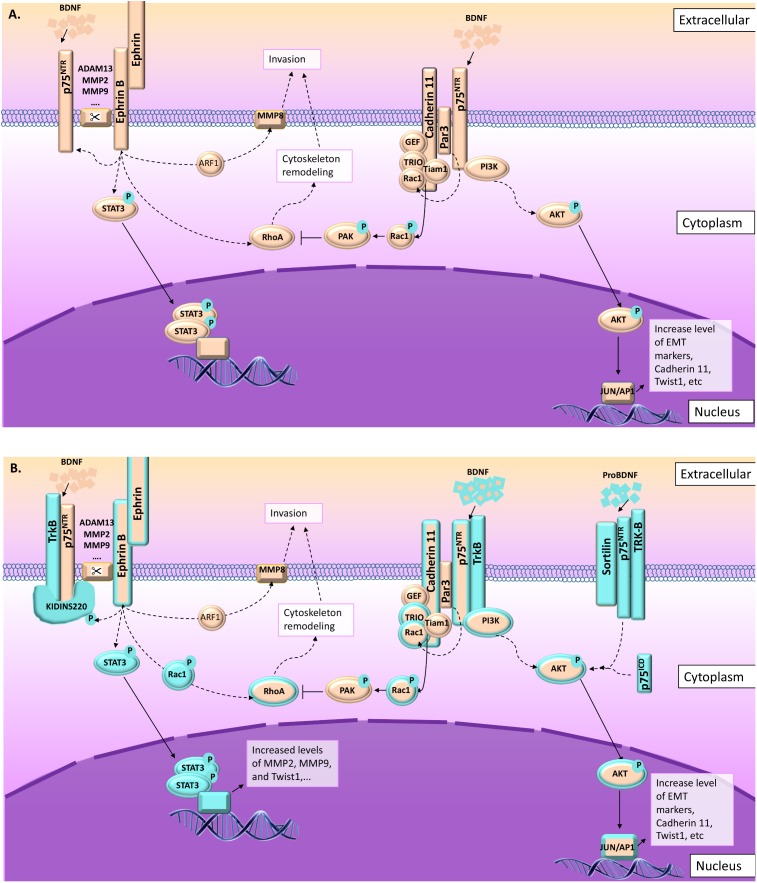
p75^NTR^ and TrkB pathways leading to migration and invasion in normal and pathological conditions. As described in this figure, several signal pathways are preferentially activated in cell invasion and migration processes involving p75^NTR^ upon NT activation. From the left to the right: (1) ephrin-B receptor phosphorylate Kidins220/ARMS leading to p75^NTR^/TrkB activation (Neubrand et al., 2012; Cai et al., 2017) and increasing their sensitivity to BDNF, leading to the activation of MAPkinase signaling pathways (Liao et al., 2011). Ephrin-B receptor activation is also linked to STAT3 and RhoA signaling leading to cytoskeleton remodeling (Marler et al., 2010; Neubrand et al., 2012). (2) In the absence of Kidins220/ARMS, p75^NTR^/TrkB could interact with other scaffold proteins like Par3 (Moore et al., 2013; Gomez et al., 2018). In this context, the BDNF activates p75^NTR^/TrkB leading to AKT pathway activation, but also to Par3 activation (Gomez et al., 2018). Activated Par3 activates Rac1, which modifies cadherin-11 interaction with catenins leading to cytoskeleton remodeling (Moore et al., 2013). (3) In some cases, p75^NTR^/TrkB also interact with sortilin leading to a high sensitivity to proBDNF. ProBDNF/p75^NTR^/TrkB also activate AKT pathway (De la Cruz-Morcillo et al., 2016). **(A)** Information collected from NCC migration and invasion during the development. **(B)** Information collected from cancer studies (highlighted in blue). Orange and blue borders = information related to both situations, NCC and cancer cells migration and invasion.

**FIGURE 4:**
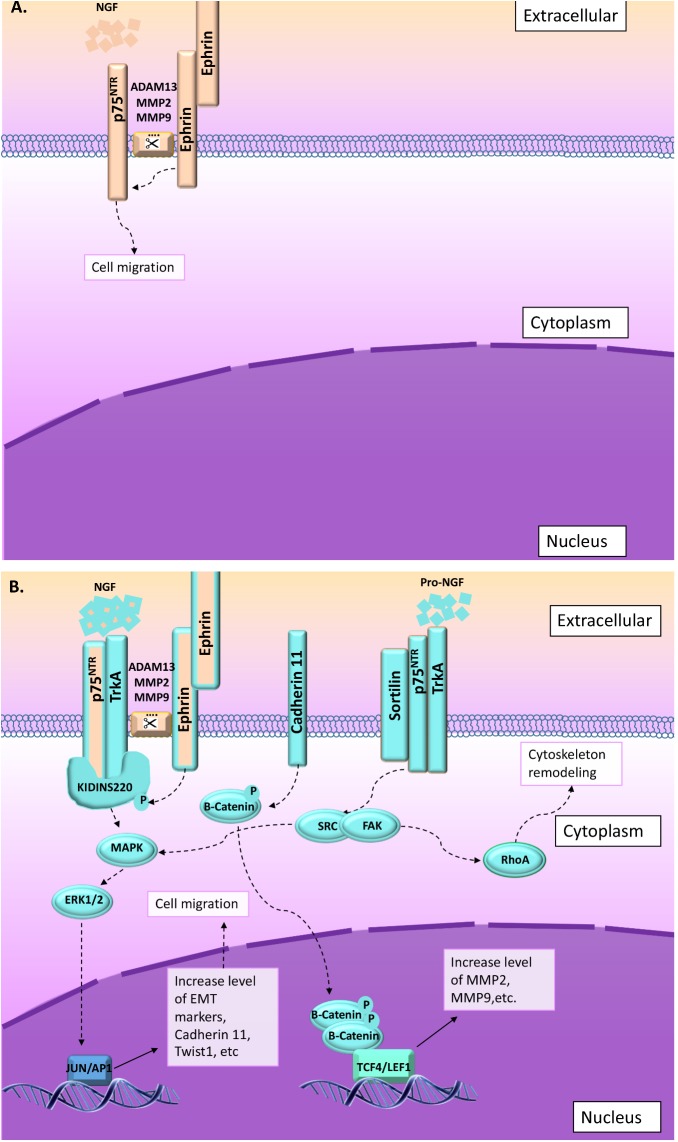
In some cases, migration and invasion are stimulated through NGF or proNGF upon activation of p75^NTR^ and TrkA. Two main pathways are described in that context: (1) ephrin-B receptor activated through interaction with ephrin-ligand (from surrounding cells) phosphorylate Kidins220/ARMS leading to increase p75^NTR^/TrkA affinity for NGF (Liao et al., 2011; Schmieg et al., 2015; Restivo et al., 2017). Upon NGF activation, p75^NTR^/TrkA/Kidins220/ARMS activate MAPkinase pathway (Liao et al., 2011). (2) Migration and invasion is also activated through proNGF/sortilin/p75^NTR^/TrkA (Faulkner et al., 2018). In this context, SRC/RhoA signaling pathway is activated leading to cytoskeleton remodeling (Faulkner et al., 2018). **(A)** Information collected from NCC migration and invasion during the development. **(B)** Information collected from cancer studies (highlighted in blue). Orange and blue borders = information related to both situations, NCC and cancer cells migration and invasion.

To understand the potential roles of p75^NTR^ in NCC migration, we have to examine further and analyze multiple molecular factors that control NCC migration (**Figure 2**). During the development, NCCs proceed to an EMT, which includes the loss of tight and adherent junctions, modifications of the apical-basal cell polarity and rearrangements of the cytoskeleton, which will help cells to start migrating (Pegoraro and Monsoro-Burq, 2013). During EMT, the transcription factor Twist-1 is activated by a variety of signal transduction pathways, including AKT signal transducers (**Figures 3A, 4A**). Activated Twist-1 upregulates *N*-cadherin and downregulates *E*-cadherin (also known as cadherin-2 and 1, respectively), which are the hallmarks of EMT (Vincentz et al., 2008). Likewise, it has been demonstrated that NCCs are trapped into the neural tube in Twist-1 knockout mice (Vincentz et al., 2008). Twist-1 is also known to induce expression of cell migration markers including periostin, cadherin-11, and MMP2/MMP9 (Shelton and Yutzey, 2008); however, it is unclear if it is through a direct or indirect induction (Kim et al., 2014).

Cadherins are proteins that could behave as ligands as well as receptors and are divided into several categories: (1) type I (classical) including *E*-cadherin (cadherin-1) and type II including *N*-cadherin (cadherin-2), as well as cadherin-11. Type I and II are ultimately linked to the actin cytoskeleton, (2) the desmosomal cadherins (desmocollins and desmogleins), which are linked to intermediate filaments, (3) the protocadherins, which are expressed primarily in the nervous system, and (4) unconventional cadherins including VE-cadherin (cadherin-5), *K*-cadherin (cadherin-6 present in kidney) and R-cadherin (cadherin-4, present in the retina) (Patel et al., 2003).

Cadherin-11 has been described as an emerging contributor to embryonic development, tissue morphogenesis, as well as tumor invasion and metastasis (Kim et al., 2014). The ectodomain of cadherin-11 leads to homophilic ligation and adhesive recognition, whereas protein interaction with the highly conserved cytoplasmic tails leads to actin cytoskeleton remodeling and cell signaling pathways (Yap and Kovacs, 2003). Cadherin-based cell adhesion activates phosphatidylinositol 3-kinase (PI3K) and AKT (Kim et al., 2014, **Figure 3A**). Likewise, it has been shown that cadherin-11 interacts with ErbB2 receptor to activate AKT pathway and NCC migration (Mathavan et al., 2017).

According to a recent study, cadherin 6 (type-2), 7 (type-2), and 12 (N-cadherin-2) are also upregulated during NCC migration, whereas cadherin-5 (VE-cadherin) and 10 (type-2) are downregulated (Morrison et al., 2017). Cadherin-6 has been suggested to promote EMT by mediating proEMT signals (Clay and Halloran, 2014). Moreover, cadherin-6 knockdown in NCC alters the subcellular distribution of active Rho, which is known to promote localized actomyosin contraction, a crucial step for apical NCC detachment (Clay and Halloran, 2014). Another study demonstrated that cadherin-7 is upregulated in migrating NCCs in response to ectopic Wnt activation (Prasad and Paulson, 2011). Likewise, Fujita et al. (2016) demonstrated that cadherin-7 directly induces FoxD3 expression when stimulated by BMP2/Wnt3a signaling and plays important roles in this process of NCC formation. The molecular pathways and the exact roles of cadherin-5, cadherin-10, and cadherin-12 during NCC migration have not been investigated so far.

Another aspect of NCC migration concerns cell collision. Collision between cells can cause them to move away from each other, a process known as contact inhibition of locomotion (CIL). CIL is required for NCC migration. As described by Smeets et al. (2016), CIL leads to regular cell arrangements and hinders the formation of cohesive tissues. CIL is a complex process that involves many different molecular mechanisms. As reviewed by Roycroft and Mayor (2016), the CIL processes can be divided into four different stages:

(1) initial contact between the cells
(2) inhibition of protrusive activity at the site of contact
(3) cell repolarization and formation of new protrusions away from the contact
(4) cell separation and migration away from each other.

Each of the four distinct steps of CIL requires changes to the cytoskeleton driven by a variety of molecular components via the recruitment of regulatory factors such as the cell polarity protein Par3 (also known as Pard3) (Moore et al., 2013; Roycroft and Mayor, 2016).

Par3 has been described as a driver for the maturation of the cell adhesion complex and has been linked to the regulation of microtubule dynamics and Rac1 activity through interaction with the Rac-GEF Tiam-1 (Moore et al., 2013, **Figure 3A**). Likewise, it has been shown that Par3 controls CIL by inhibiting the Rac-GEF Trio to prevent Trio-mediated activation of Rac1 at cell–cell contacts (Moore et al., 2013, **Figure 3A**). Nonetheless, Moore and collaborators suggested that cadherin 11 act as a regulator of Par3 functions at the cell contact as it has been shown that during NCC migration, the Rac-GEF Trio interact with cadherin 11, which is localized to cell protrusions as well as cell-cell contacts (Moore et al., 2013, **Figure 3A**). The link between Par3 and p75^NTR^ has not been established during NCC migration; however, it has been demonstrated that in Schwann cell myelination, BDNF activates Par3 through p75^NTR^ interaction in order to induce Rac1 (Tep et al., 2012, **Figure 3A**).

Besides Par3, the switch from *E* to *N*-cadherin, as well as from ephrin A to ephrin B are essential for CIL between NCCs (Roycroft and Mayor, 2016). Again cadherin-11 is implicated in CIL since Becker et al. (2013) demonstrated that cadherin-11 mediated cell–cell adhesion was necessary in CIL for directional and collective NCC migration through Rac1 and small Rho-GTPase RhoA signaling. Indeed, Rho-GTPases play active roles in remodeling the actin cytoskeleton and in the regulation of microtubules (Roycroft and Mayor, 2016). Interestingly, it has been demonstrated that the DD of p75^NTR^ is able to interact with Rho-GDI for the activation of RhoA pathway (Lin et al., 2015, **Figure 3A**).

Ephrins are membrane-bound proteins that mediate bidirectional signals between adjacent cells (Baker and Antin, 2003). Ephrins are known to direct cell movements during multiple morphogenetic processes, including NCC migration, through cytoskeleton remodeling processes (Baker and Antin, 2003). Ephrin receptors and ephrin ligands are subdivided into two subclasses with distinct binding specificities that correlate with structural similarities: Ephrin-A ligands (named ephrin-A1 to ephrin-A5) are anchored in the plasma membrane through a GPI-linkage, and each of them can bind any of the ephrin-A subclass of receptors (ephrin-A1 to ephrin-A8) (O’Leary and Wilkinson, 1999). On the other hand, ephrin-B ligands (ephrin-B1 to ephrin-B3) have a transmembrane domain and a cytoplasmic region and are able to interact with members of the ephrin-B subclass of receptors (ephrin-B1–ephrin-B6) as well as to the ephrin-A4 receptor (O’Leary and Wilkinson, 1999).

As reviewed by Theveneau and Mayor (2012), migratory NCCs and their surrounding tissues express a wide variety of ephrins. Indeed, ephrin signaling takes place in three different situations:

(1) NCCs belonging to different streams express different genetic repertoires, preventing them from mixing and keeping the streams separated (Theveneau and Mayor, 2012).

(2) NCCs are prevented from invading areas where non-NCCs express complementary ephrins (Theveneau and Mayor, 2012).

(3) NCCs and their invaded tissues may display matching receptor and ligand codes to allow homing (Theveneau and Mayor, 2012).

Ephrin-A4 and ephrin-A5 have been described as downregulated during early NCC migration, whereas ephrin-B1 was upregulated (Morrison et al., 2017). Ephrin-B1 has been reported to interact with the signal transducer and activator of transcription 3 (STAT3), in a phosphorylation-dependent manner that leads to enhanced activation of STAT3 transcriptional activity (Bong et al., 2007, **Figure 3A**). Moreover, it was demonstrated that STAT3 activity, in this context, depended on the tyrosine kinase Jak2, and two tyrosines within the ICD of ephrin-B1 that were critical for its association with STAT3 and its activation (Bong et al., 2007).

A closer look to ephrin receptors and ligands indicated that their interactions occur between adjacent cells mediating juxtacrine signaling (Atapattu et al., 2014). Ephrin receptor and ligand interactions involve clusters of receptors and ligands leading to large receptor–ligand complexes, also including cadherins (Atapattu et al., 2014). Ephrin activation requires proteolytic cleavage that is processed by a variety of proteases including a disintegrin and metalloproteases (ADAMs) (Atapattu et al., 2014, **Figures 3A, 4A**). ADAM13 has been reported to specifically cleave ephrin-B ligands as well as other proteins including cadherin-11 and is linked to NCC migration during the development (Gaultier et al., 2002). In this context, it appears that ADAM13 is able to upregulate canonical Wnt signaling and early expression of the transcription factor snail2, whereas ephrin-B inhibits this pathway, suggesting a role of ephrin-B cleavage by ADAM13 in the repression of canonical Wnt signaling (Wei et al., 2010).

Similar to ADAM proteases, matrix metalloproteases (MMP) have been reported to cleave ephrin ligands and receptors. MMPs are classically described for their ability to degrade extracellular matrix components to facilitate cell migration; however, they also participate in the proteolysis of several growth factors and receptors like FGFR or ErbB receptors (Peschon et al., 1998). Looking at cell migration and invasion, it has been reported that ephrin-B2 receptor which is activated upon ephrin-B1 ligand interaction and MMP8 cleavage, activate Arf1 and RhoA allowing their interacting with Disheveled and leading to cell repulsion and invasion (Lin et al., 2008, **Figure 3A**). Likewise, MMP2/MMP9 have been described as activating ephrin-B4 receptor/ephrin-B2 ligand and mediate migration of endothelial cells via PI3Kinase/AKT pathway (Steinle et al., 2002). As mentioned above, Twist-1 is a regulator of MMP2/MMP9 expression leading to early NCC migration, during the development (**Figure 3A**).

Regarding p75^NTR^ interactions with ephrins, it has been shown that the BDNF induced ephrin-A5 ligand/eprhin-B2 receptor/p75^NTR^ interactions leading to cytoskeleton remodeling (Marler et al., 2010, **Figure 3A**). Another interesting link between p75^NTR^ and ephrin receptors and/or ligands has been established several years ago as kinase D-interacting substrate of 220 kDa/ankyrin repeat-rich membrane spanning (Kidins220/ARMS), a scaffold protein complex, has been described as interacting with p75^NTR^ as well as other receptors, including ephrin receptors (Neubrand et al., 2012). Most importantly, it was shown that Kidins220/ARMS was activated by phosphorylation after treatment with NGF (**Figure 4A**), BDNF (**Figure 3A**), and ephrin-B2, suggesting a critical link between cell surface receptors and intracellular signaling events for both the NT and the ephrin families (Kong et al., 2001). However, the link between NT, p75^NTR^, and ephrin in migrating NCCs has not been described so far.

Altogether, it appears that during NCC invasion and migration, p75^NTR^ may play several roles in EMT and CIL events through several pathways including AKT, Rho-GTPase RhoA, and Jak2/STAT3. These signaling pathways seem to be mainly activated through interactions with ephrin and cadherin family members as summarized in **Figures 3A, 4A**, and could modulate NT effects through p75^NTR^ and potentially coreceptors as well as through scaffold proteins. However, even if ephrin, cadherin, along with p75^NTR^, BDNF, and NGF have been implicated in NCC migration and invasion during the development, it is still unclear which coreceptors and/or effectors are involved in such processes. To better understand and “fill the gaps,” we will now analyze our actual knowledge about p75^NTR^ roles in cancer cell migration and invasion.

## Roles of p75^NTR^ in Cancer Cell Migration and Invasion

Cancer stem cells (CSCs), also known as tumor-initiating cells, represent a minority population of tumor cells that share the biological characteristics of normal stem cells such as self-renewal and differentiation (Suraneni and Badeaux, 2013). Moreover, CSCs are described as resistant to conventional therapies and are thought to be responsible for recurrence as a driving force for tumor initiation, recurrences, or metastasis. Their identification is functional or operational as they are the only tumor cells able to start a tumor in a xenograft procedure and they are able to grow as spheres when cultivated. Indeed, at least so far, there is no specific marker to identify CSCs.

As mentioned above, during the development, NCCs proceed to an EMT that includes Twist-1 activation leading to the switch from *E* to *N*-cadherin (Pegoraro and Monsoro-Burq, 2013). These changes are highly similar to those observed in metastatic tumor cells undergoing EMT (Pegoraro and Monsoro-Burq, 2013), as well as in circulating tumoral cells (CTCs) (Lombard et al., 2015). In several cancers, Twist-1 has been identified as a master regulator of EMT (Firulli and Conway, 2008) and is characterized by a downregulation of *E*-cadherin and an upregulation of *N*-cadherin on cell surfaces (Cho et al., 2016). Twist-1 plays an important role in some physiological processes involved in metastasis. In addition, Twist-1 has been described as being responsible for the maintenance of CSCs, the development of chemotherapy resistance (Khan et al., 2013), and in some instances, the radiotherapy resistance (Xiong et al., 2017). In this context, STAT3 signaling pathway seems to be the most common pathway that targets genes like MMP-2, MMP-9, VEGF, and Twist1, which are involved in cell migration and invasion (Cao et al., 2016, **Figure 3B**).

Another remarkable common point with NCC migration and invasion during development is the fact that cadherin-11 has also been reported to be overexpressed in several cancers and linked to cell migration and invasion (**Figures 3B, 4B**). Indeed, Birtolo et al. (2017) demonstrated that the knockdown of cadherin-11, in cancer cells, reduced pancreatic cancer cell migration. Likewise, Satcher et al. (2015) showed that clatherin-mediated internalization of cadherin-11 regulated the surface trafficking of cadherin-11 and that the dynamic turnover of cadherin-11 regulated the migratory function of cadherin-11, in prostate cancer cells. It was also suggested that p75^NTR^ could impact U87 glioblastoma cell migration through modulation of specific genes, including cadherin-11 (Berghoff et al., 2015). Finally, Li et al.’s (2011) study revealed a novel cadherin-11-Trio-Rac signaling axis that contributes significantly to breast cancer cell migration (**Figure 3B**). As mentioned above, during NCC migration, cadherin-11 modulate the Par3 function that has been linked to the regulation of microtubule dynamics by inhibiting the Rac-GEF-Trio to prevent Trio-mediated activation of Rac1. Likewise, the BDNF has been shown to activate Par3 through its interaction with p75^NTR^. In cancer, studies that performed genome-wide screening for microdeletions revealed that the region containing the Par3 gene was deleted in lung, head and neck, and esophageal squamous cell carcinoma cell lines (Nakamura et al., 2016). In those cancers, Par3 seems to act as a tumor suppressor. Likewise, Par3 has been described as a metastasis inhibitor in pancreatic and breast cancer; however, it has also been shown that in those two cases that Par3 presents an alternative splicing, which could change its interaction capacities with target factors including Rac1 (Ke et al., 2018). On the other hand, in clear cell renal carcinoma, Par3 overexpression was associated with poor prognosis (Nakamura et al., 2016). Likewise, in skin cancer, Par3 acts as a tumor suppressor or a tumor promoter depending on the tumor type; in keratoacanthoma it acts as a tumor suppressor, whereas it acts as a tumor promotor for melanoma (Nakamura et al., 2016). Interestingly, abnormal NT signaling has been implicated in the progression of numerous cancers, including neuroblastoma, medulloblastoma, melanoma, papillary thyroid carcinoma, pancreatic cancer, prostate cancer, and breast cancer (Molloy et al., 2011). Indeed, in lung cancer (Gomez et al., 2018), head and neck cancer (Jiang et al., 2017), and esophageal squamous cell carcinoma (Gomez et al., 2018), BDNF/TrkB/PI3K-AKT seem to be activated and linked to invasiveness (**Figure 3B**). As mentioned above, BDNF/p75^NTR^/PI3K-AKT activate Par3 blocking the Par3 effect (as a negative regulator) on RhoA pathway leading to cytoskeleton remodeling and therefore inducing invasiveness. In clear cell renal carcinoma, it has been shown that invasiveness is independent of BDNF/TrkB signaling, but dependent on the high expression level of p75^NTR^ working through proBDNF activation (De la Cruz-Morcillo et al., 2016, **Figure 3B**). So far, the link between proBDNF and Par3 has not been established; however, it is possible that proBDNF may indirectly act on Par3 leading to an opposite effect to the one described for the BDNF, similar to what we observed for p75^NTR^. Likewise, it was also shown that proBDNF induced cell migration through p75^NTR^/TrkB/sortilin leading to the activation of AKT and ERK pathways (De la Cruz-Morcillo et al., 2016, **Figure 3A**). In melanoma, a recent study characterized the interaction of NGF/p75^NTR^/TrkA signaling as a regulator of phenotype switching in melanoma (Restivo et al., 2017, **Figure 4B**). In this study, the authors described p75^NTR^ as a key effector to drive the melanoma cells to become highly invasive (Restivo et al., 2017). Likewise, in thyroid cancer cells, proNGF works through p75^NTR^/TrkA/sortilin leading to SRC signaling pathway activation and cell invasion (Faulkner et al., 2018, **Figure 4B**).

Beside Par3, other CIL contributors are also necessary for cancer progression. Indeed, it has been described that ephrin-B signaling gives rise to CIL in carcinoma cell lines and can induce high levels of CIL behavior (Roycroft and Mayor, 2016). Likewise, it was demonstrated that ephrin-B4 receptor signaling contributes to the high migratory ability of invasive melanoma cells by influencing RhoA-mediated actin cytoskeleton reorganization (Yang et al., 2006, **Figure 3B**). Similarly, ephrin-B2 and ephrin-B3 expression and phosphorylation correlated with increasing tumor grade and its signaling through Rac1 was critically important to glioma invasion (Nakada et al., 2006, 2010, **Figure 3B**).

Currently, the molecular pathways underlying the role of p75^NTR^ in cancer cell migration and invasion is not fully understood; however, multiple clues in several cancers linked p75^NTR^ to tumor cell migration and invasion. Indeed, proBDNF has been described as inducing renal cell carcinoma cell survival and migration, through p75^NTR^ (De la Cruz-Morcillo et al., 2016). In the same line, proNGF is associated with the invasion and migration of melanoma cells through a mechanism involving p75^NTR^ and sortilin (Truzzi et al., 2008). Likewise, Shonukan et al. (2003) showed that the activation of p75^NTR^ with NGF or proNGF induced melanoma cell migration and increased the level of expression of p75^NTR^ (Shonukan et al., 2003). Moreover, the effects of NGF and proNGF through p75^NTR^ were correlated with the late stages and the invasive potential of melanoma brain metastasis (Shonukan et al., 2003, **Figure 4B**).

p75^NTR^ was also correlated with a poor prognosis in human hypopharyngeal cancer (HPC) as Mochizuki and collaborators indicated that p75^NTR^ initiated tumor formation by the activation of Erk-signaling leading to an acceleration of the migration signaling pathway (Mochizuki et al., 2016, **Figure 4B**). Likewise, in esophageal squamous cell carcinoma, p75^NTR^ overexpression and PI3K/AKT signaling pathway activation induced invasion and migration (Xi et al., 2016, **Figure 3B**). Likewise, Gomez et al. (2018) demonstrated that in esophageal squamous cell carcinoma, invasiveness was linked to BDNF/TrkB/PI3K-AKT (**Figure 3B**).

In glioma, several cell lines characterized as brain tumor-initiating cells (BTIC) have been established by culturing cells derived from patient tumors. Using those cell lines, several studies demonstrated that the NGF stimulates BTIC proliferation through a p75^NTR^ cleavage. Interestingly, ectopic expression of p75^NTR-ICD^ was sufficient by itself to stimulate BTIC cell invasion and proliferation (reviewed by Chopin et al., 2016). Moreover, p75^NTR-ICD^ was able to induce AKT activation in BTIC (Forsyth et al., 2014). Likewise, Berghoff and collaborators also indicated that p75^NTR^ undergoes a γ-secretase-mediated regulated intramembrane proteolysis and was involved in glioblastoma cell migration and invasion (Berghoff et al., 2015). More recently, Alshehri and collaborators indicated that p75^NTR^ was a central regulator of glioma invasion (Alshehri et al., 2017). These observations correlate with a study indicating that PDLIM1, a novel signaling adaptor for p75^NTR^, was shown to interact with p75^NTR^ in highly invasive patient-derived glioma stem cells/tumor-initiating cells (Ahn et al., 2016). Moreover, silencing PDLIM1 (a member of the PDZ and LIM protein family) *in vitro* and *in vivo* resulted in a complete ablation of p75^NTR^-mediated invasion (Ahn et al., 2016).

In medulloblastoma (MB), the most aggressive brain tumor in children, it has been reported that p75^NTR^ expression is correlated with cell invasion and migration (Wang et al., 2015). Indeed, in human MB cell lines, p75^NTR^ was shed by α-secretase first to generate ECD and the carboxy-terminal fragment, which was still anchored in the membrane, was then cleaved by γ-secretase to generate an ICD. This p75^NTR^ proteolytic processing was required for p75^NTR^-mediated MB invasion *in vitro* and *in vivo* (Wang et al., 2015, **Figure 3B**).

All these cancer studies revealed a strong implication of p75^NTR^ in cell migration and invasion that seems to be induced through multiple pathways. This observation is even reinforced by the fact that besides NTs and coreceptors that have been linked to migration and invasions, other effectors may also induce cell migration and invasion through p75^NTR^. In fact, cell migration and invasion have also been reported to be activated by p75^NTR^ through a protein scaffold like Kidins220 or a p75^NTR^ modulator like NRAGE.

Currently, there is growing evidence showing the involvement of Kidins220/ARMS in various cancers (Raza et al., 2017). As mentioned above, Kidins220/ARMS is a multifunctional transmembrane scaffold protein involved in the regulation of many cellular functions. The most significant role identified for Kidins220/ARMS is as a downstream substrate of NT receptors (Cai et al., 2017). Kidins220 appeared to be phosphorylated following exposure to ephrin-B, suggesting a role downstream of ephrin receptors (Cai et al., 2017). Kidins220/ARMS has also been reported to mediate melanoma cell migration and invasion through activation of ERK/MEK signaling pathways (Liao et al., 2011, **Figure 4B**). Moreover, the NGF and the BDNF have been shown to modulate the Kidins220/ARMS expression level (Schmieg et al., 2015) and its overexpression drastically induced TrkA expression (Schmieg et al., 2015). As mentioned above, TrkA and p75^NTR^ overexpression have been linked to migration of several cancer cells like in thyroid cancer (Faulkner et al., 2018) or in pancreatic cancers (Bapat et al., 2016). As Kidins220/ARMS is also able to interact with TrkB and TrkC, it is possible that Kidins220/ARMS overexpression could also modulate the TrkB and TrkC level of expression depending on the type of NT induction; however, it has not been investigated so far (**Figure 3B**).

Similarly to Kidins220/ARMS, numerous efforts have been made to dissect the relationship between NRAGE and tumorigenesis (Zhang et al., 2016). NRAGE also known as MAGE-D1 or Dlxin-1 plays crucial roles in regulating tumorigenesis and metastasis, as its downregulation is associated with metastasis formation in a variety of tumor cells including pancreatic cancer, low-grade gastric cancer, and ovarian cancer (Zhang et al., 2016). NRAGE is known to inhibit cell migration through its interaction with *E*-cadherin. Indeed, *E*-cadherin is known as a cell–cell adhesion molecule that inhibits motility through its interactions with α-catenin, β-catenin, and p120-catenin as well as with ankyrin-G (Cai et al., 2014). The α-catenin and β-catenin link also the actin filaments of the cytoskeleton. When the interactions between *E*-cadherin and catenin are broken, β-catenin is released in its phosphorylated state. Phosphorylated β-catenin is then transferred from the cytoplasm into the nucleus, where it interacts with transcription factors. Ankyrin-G is known to interact with the cytoskeleton through spectrin and actin complexes and with the plasma membrane, through diverse transmembrane proteins including *E*-cadherin (Frisch et al., 2013). Ankyrin-G has also been shown to sequester NRAGE in the cytoplasm (Kumar et al., 2011). In the absence of ankyrin-G (i.e., after the loss of *E*-cadherin following EMT), a fraction of NRAGE translocates to the nucleus, where it has been shown to interact with the oncogenic transcriptional repressor protein TBX2 (Kumar et al., 2011). TBX2 has been described as a strong inducer of EMT leading to tumor progression from non-invasive to invasive malignant states (Wang et al., 2012).

So far, NRAGE overexpression has been described as playing a role in migration and invasion of breast cancer (Wang et al., 2012), hepatocellular carcinoma (Shimizu et al., 2016), and high-grade gastric cancer (Kanda and Kodera, 2016). As previously mentioned, in breast cancer, cell migration and invasion are linked to TrkA/ERK/p38 MAP kinase pathways (Lagadec et al., 2009, **Figure 4B**). Likewise, cadherin-11/p75^NTR^/Rho-A and Par3 alternative splicing seem to be also implicated in breast cancer migration and invasion (**Figure 3B**). On the other hand, in gastric cancer, p75^NTR^ has been reported to inhibit the invasive and metastatic abilities of low-grade cancer cells by suppressing the NF*kappa*B signal transduction pathway potentially through p75-ICD and NRAGE interaction (Jin et al., 2007). However, in high-grade gastric cancer, cellular migration and invasion are mediated through EZH2, COX-2, MCL-1, and FOS genes. Interestingly, EZH2 (the polycomb repressor complex 2 molecule) is known to interact with the TrkA promotor and modulate TrkA silencing (Li et al., 2018).

Finally, it appears that the level of expression of p75^NTR^ would have different effects depending on the cell types. On one hand, its overexpression seems to induce apoptosis in colorectal cancer (Yang et al., 2014) and block the cellular cycle in liver cancer (Yuanlong et al., 2008). On the other hand, p75^NTR^ promotes survival in breast cancer or metastasis formation and cell invasion in glioma and melanoma (reviewed by Chopin et al., 2016). Interestingly, all cancers in which p75^NTR^ appears to have a repressive effect come from the mesoderm or the endoderm (Chopin et al., 2016). On the contrary, the effects linked to a poorer vital prognosis through an increase rate of metastatic formation, invasion, and migration are observed in cell types arising from the (neur)ectoderm (Chopin et al., 2016). This apparent simple observation is actually even more complicated than suggested above since in low-grade glioma, p75^NTR^ and Trk coreceptors have been reported to inhibit tumor growth and survival, whereas the opposite effects were reported in high-grade gliomas (Alshehri et al., 2017). In low-grade gliomas, it appears that the glial cell-derived neurotrophic factor (GDNF) promotes cell migration through JNK, ERK-1/2, and p38 MAPK signaling pathways (Song and Moon, 2006). Likewise, the levels of expression of the NGF and its high-affinity receptor (TrkA) have been shown to be reduced in low-grade gliomas, whereas that of p75^NTR^ was increased (Chiaretti et al., 2004). In high-grade gliomas, Xiong and collaborators demonstrated that the BDNF promoted cell migration and invasion through TrkB and p75^NTR^, whereas proBDNF had the opposite effects (Xiong et al., 2013). Similar observations were made for other cancers as an increased level of TrkB and BDNF as well as the downregulation of expression of E-cadherin were correlated with the invasion and metastasis of salivary adenoid cystic carcinoma cells (Jia et al., 2015). In gastric cancer, p75^NTR^ has been reported to inhibit the invasive and metastatic abilities of cancer cells in correlation with the downregulation of metalloprotease-9 (MMP9) proteins and upregulation of the tissue inhibitor of matrix metalloprotease-1 (TIMP1) protein by suppressing the NF*kappa*B signal transduction pathway (Jin et al., 2007).

All of those observations suggest that p75^NTR^ effects seem to be independent on the cell type, but those effects are rather modulated by the type and the level expression of the specific coreceptor (Trk, sortilin, and Nogo/LINGO) or effector (Par3, Kidins220 scaffold proteins, NRAGE modulator, ephrin, and cadherin) as well as a fine balance between NT and proNT. All these receptors/effectors are able to modulate the signaling pathways induced by extracellular factors including NTs, leading to an array of roles that could be finely tuned.

## Conclusion

**FIGURE 5:**
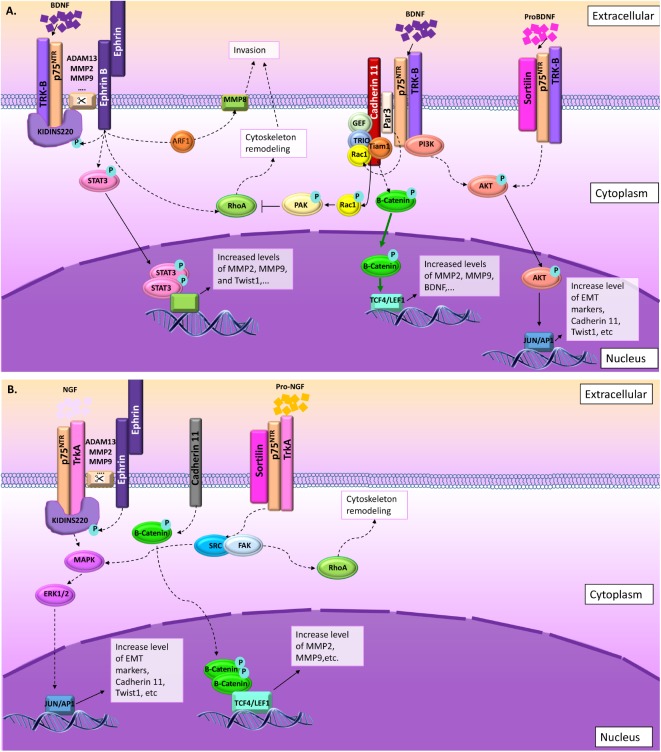
Summary of the signaling pathways involved in cell migration and invasion through p75^NTR^. **(A)** BDNF NT may act through p75^NTR^/TrkB/Kidins220 following ephrin receptor interaction and phosphorylation or through p75^NTR^/TrkB/Par3 following cadherin interactions. In some rare cases, proBDNF may also induce migration and invasion through sortilin/p75^NTR^/TrkB interactions. **(B)** In the presence of NGF, p75^NTR^/TrkB/Kidins220 following ephrin receptor interaction and phosphorylation induce MAPK/ERK signaling pathway. Again, in some rare cases, proNGF may also induce migration and invasion through sortilin/p75^NTR^/TrkA interactions leading to SRC/FAK activation.

Looking specifically at the implication of p75^NTR^ in cell migration and invasion in normal and pathological conditions, it appears that two main pathways seem to be mainly activated: (1) BDNF/p75^NTR^/TrkB (**Figure 5A**) and (2) NGF/p75^NTR^/TrkA (**Figure 5B**). Depending on the type of effectors, different signaling pathways may be activated, leading to the same migration and invasion effect. In some cases, proNTs (proNGF and proBDNF) may replace the NT effect through p75^NTR^ and sortilin activation (**Figures 5A,B**). From NCC migration and invasion, we learned that ephrin and cadherin are two important groups of membrane proteins that regulate mechanisms of cell invasion and migration through p75^NTR^. However, signaling pathways involved in such processes are still unknown. What we could suggest from cancer cell migration studies is that in the presence of the BDNF, p75^NTR^ may be activated by TrkB and Kidins220 upon ephrin receptor phosphorylation leading to STAT3 activation and RhoA inhibition. Likewise, in the presence of the NGF, p75^NTR^/TrkA/Kidins220 may be activated upon ephrin receptor phosphorylation leading to MAPK/ERK pathway activation. Likewise, nothing is known about proNGF or proBDNF and sortilin effects on NCC migration and invasion.

Moreover, even if multiple clues implicate p75^NTR^ in cancer cell migration, questions regarding the exact roles of this receptor are still numerous. The first question could be related to the mode of regulation of p75^NTR^ functions. During migration, in normal and pathological conditions, p75^NTR^ could act through several pathways including direct interaction with neurotrophins, modulation of NT affinity of other receptors through a direct or indirect contact with those receptors, and through the release of ECD or ICD upon secretase cleavages. The second question relates to the origin of external factors like NTs that will activate p75^NTR^. Are those factors present in endogenous normal cells or only in modified pathological environments? All these questions appear more and more pertinent and would require an urgent response as they could pave the way to set up new targeted therapeutic approaches.

## Author Contributions

SW conceived and designed the presented idea and provided administrative support. SW and BR provided financial support. SW, GV, and BR contributed to the writing of the manuscript.

## Conflict of Interest Statement

The authors declare that the research was conducted in the absence of any commercial or financial relationships that could be construed as a potential conflict of interest. The reviewer RU and handling Editor declared their shared affiliation at time of review.
